# Landslide investigations during pandemic restrictions: initial assessment of recent peat landslides in Ireland

**DOI:** 10.1007/s10346-021-01797-0

**Published:** 2021-12-30

**Authors:** Alan P. Dykes

**Affiliations:** Centre for Engineering, Environment and Society Research, School of Engineering and the Environment Kingston University, Penrhyn Road, Kingston upon Thames, KT1 2EE UK

**Keywords:** Upland blanket peat, Bogflow, Bog slide, Peat landslide, Ireland

## Abstract

Landslides involving peat are relatively common in Ireland, upland areas of Great Britain and subantarctic islands. Bogflows and bog slides are less common types of peat failure and almost unknown outside Ireland. Unusually, three of these occurred in 2020 including one bogflow at a windfarm that gained much adverse media attention, and a small but damaging peat slide was also reported. The aim of this paper is to determine the extent to which the new bog slide and bogflows are consistent with previous examples in terms of their contexts, characteristics and possible causes, particularly relating to commercial forestry operations. Aerial video footage of all three landslides obtained by local people using drones, and ground-based footage of one of them in progress, allowed a detailed examination of their characteristics and contexts to be made despite the global travel and activity restrictions caused by the coronavirus pandemic. The windfarm bogflow appears to have resulted from removal of toe support by an earlier peat flow that was itself probably caused by construction of an access road; the other two landslides were most likely triggered by rainfall. All three are consistent with previous examples of their respective types in their general characteristics and appear to be associated with well-known causal factors including hydrological, topographic and/or forestry influences. Forestry operations probably contributed to the occurrence of two of the landslides and restricted the expansion of two of them.

## Introduction

Landslides involving peat have become well known as geomorphological and geotechnical phenomena since a series of damaging events in 2003 in western Ireland and northern Scotland (Lindsay and Bragg 2005; Long and Jennings 2006; Dykes and Warburton 2007a, 2008). Previously sporadic reports of specific individual events started to be collated and re-assessed around that time to start to identify common causal factors and, thus, potential risk factors (Warburton et al. 2004; Dykes and Kirk 2006; Dykes 2008a; Boylan et al. 2008). As part of this rapid expansion of new knowledge of the topic, Dykes and Warburton (2007b) presented a formal classification scheme intended to reduce further confusion arising from inconsistent use of descriptive names in the literature. Some types of failure (bog slides, peat slides, peaty-debris slides and at least some peat flows) involve shearing and sliding on a discrete failure surface but the precise mechanism of failure in bog bursts and bogflows is as yet unknown. The distinction between bogflows (with bog bursts) and bog slides is therefore made according to field evidence of failure morphology and the presence or absence of an observable failure surface.

Although relatively common in many parts of the British Isles and on several subantarctic islands with climates similarly favourable for the formation of blanket peat (Lindsay et al. 1988), few failures of peat deposits are known from elsewhere in the world (Dykes and Selkirk-Bell 2010). Many peat failures occur in more remote locations where they may pose relatively little hazard to local communities and associated infrastructures, but they are often locally environmentally damaging because the more highly humified (decomposed) peat is a strong pollutant to aquatic life where runout sediment enters streams and lakes (e.g. McCahon et al. 1987; Wilson et al. 1996). In some past cases, the runout affected watercourses, agricultural land and even treated water supplies to whole towns many kilometres downstream (Dykes and Jennings 2011). Furthermore, they result in the fragmentation, exposure, oxidation and associated release of carbon dioxide from sometimes very large quantities of what is one of the Earth’s most effective carbon-storing materials.

**Fig. 1:**
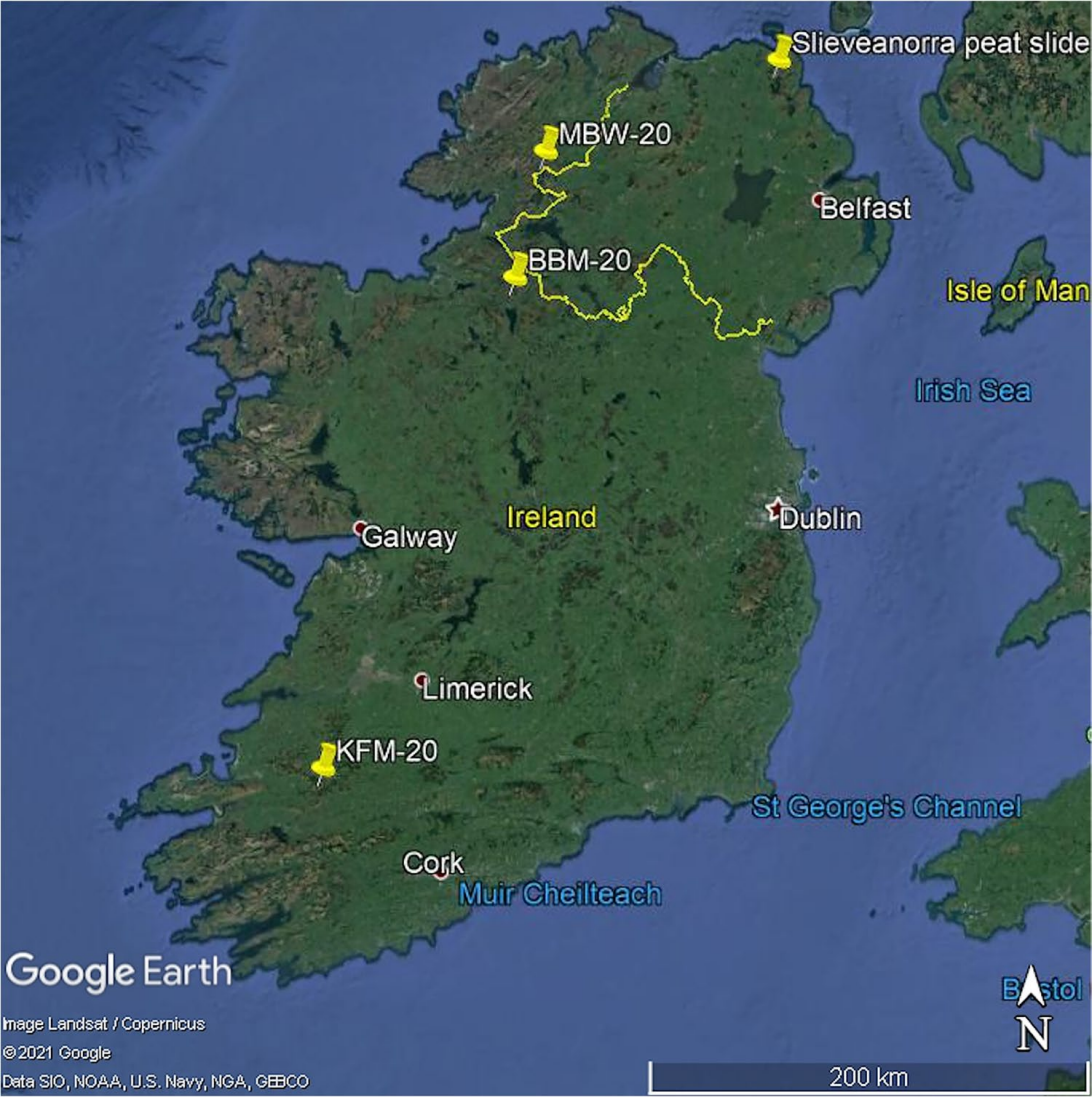
Locations of the five peat landslide events of 2020 in Ireland (two at ‘MBW-20’). Source: Google Earth (Image Landsat/Copernicus; Data SIO/NOAA/U.S. Navy/NGA/GEBCO; ©2021 Google)

Following several years with only a handful of significant peat failures, four events thought to comprise five landslides occurred in 2020 (Fig. 1) and were initially reported by Petley (2020 and subsequent posts):

1. In the evening of Sunday 28 June 2020, a large bogflow occurred on Boleybrack Mountain near Lough Allen, Co. Leitrim, Republic of Ireland. This landslide (Ref. ‘BBM-20’ in this paper) is notable for being the first of its type to occur for several years, and the largest of its type since the Glendun bogflow of 1963 (Colhoun et al. 1965).
2. On 25 August, a small landslide of around 2000–3000 m^3^, provisionally identified as a peat slide, was triggered by heavy rainfall from Storm Francis on the northern spur of Slieveanorra, Co. Antrim, Northern Ireland, at 55°05ʹ28ʺN, 6°13ʹ59ʺW (Ordnance Survey Ireland grid coordinate D 129 286). It blocked the same road as on previous occasions (e.g. Tomlinson and Gardiner 1982) but in this case the disruption was short-lived.
3. On 13 November, a bogflow occurred at Meenbog in Co Donegal, Republic of Ireland (Ref. ‘MBW-20b’ in this paper). This landslide is particularly interesting because of its relationship to construction of a new windfarm and a preceding failure (‘MBW-20a’).
4. Sometime shortly before the Meenbog event, a significant landslide—provisionally classified as a bog slide—occurred on the forested northern slope of Knockanefune Mountain in Co Kerry, Republic of Ireland (Ref. ‘KFM-20’). Here, the forestry context demands specific consideration.

This Technical Note examines the degree to which the recent bogflows (BBM-20, MBW-20b) and bog slide (KFM-20) have characteristics and causal factors consistent with previous such landslides for which details are known, and assesses the possible role of commercial forestry as a contributory causal factor in each case. The peat slide (No. 2, above) is not included in this study because peat slides and peaty-debris slides are more common and generally much smaller. The purpose of the study is to enhance knowledge and awareness of risk factors for which future provisions may need to be made by landowners and relevant authorities, given the scale of any potential direct environmental and indeed economic impacts of such events.

## Materials and methods

All of the recent landslides reported in this paper occurred during the global coronavirus pandemic when travel and other restrictions prevailed. This work was made possible by the existence and availability of aerial video footage of the three large landslides obtained by local people using drones, but also some unique ground-based footage of one of them in progress. Because of the inadvisability and effective impossibility of travelling to undertake fieldwork, all details and characteristics of these failures have been derived from three sources: (a) Google Earth (hereafter referred to as ‘GE’); (b) Ordnance Survey of Ireland (‘OSI’) 1:50,000 scale ‘Discovery Series’ topographic maps; and (c) the third-party video footage made available on-line on YouTube or Twitter (for which permission to use here was readily granted). Characteristics of all reported bogflows (*n* = 17) and bog slides (*n* = 20) since 1895 were obtained from published accounts, in many cases supplemented by more recent field examinations (e.g. Dykes 2009). These records allow some assumptions to be made about the new landslides, including displacement of the full thickness of peat across large parts of the source areas following failure somewhere within typically the lowest 0.5 m. Source area gradients of the 2020 landslides were derived from the 1:50,000 maps. As such, this study demonstrates the present feasibility of one possible ‘future’ mode of landslide research—i.e. undertaken entirely from the office—expounded by Brunsden (1993).

## Details of the recent landslides

### BBM-20: Boleybrack Mountain bogflow

#### Site characteristics and trigger conditions

**Fig. 2:**
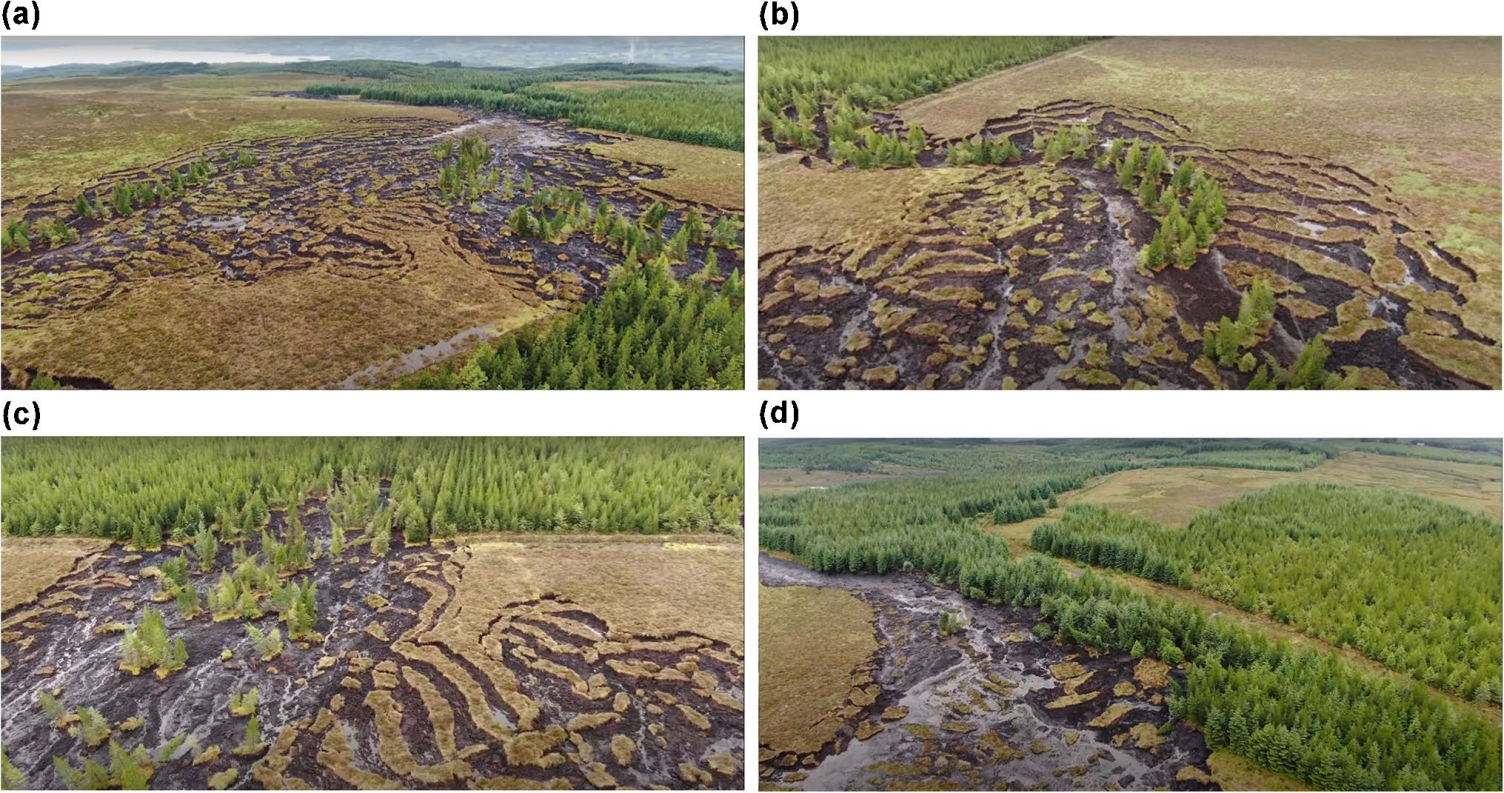
**a** The source area of the Boleybrack Mountain bogflow, looking approximately towards SSW. The ‘main’ head is at the extreme right within the forest. Source: 2 m 39 s in Flynn (2020). **b** Train of trees heading south from the secondary head of the bogflow, with adjacent peat subsiding inwards towards an apparent subsurface drainage line. Source: 1 m 03 s in Flynn (2020). **c** The main head of the bogflow with much greater evacuation of peat and associated transport of rafts of upper peat. In **b** and **c,** note how the transported trees remain upright as the peat rafts effectively float on top of the flow of semi-liquid lower peat. Source: 1 m 20 s in Flynn (2020). **d** The toe of the source area looking downstream. Note the left margin has been eroded, with some rafts pushed up against it, whilst on the right some trees have been pushed over by the moving mass of peat. Source: 3 m 05 s in Flynn (2020)

The source area of this bogflow is roughly centred on 54°12ʹ30ʺN, 8°04ʹ40ʺW (OSI grid coordinate G 948 285), 6 km northeast of Drumkeeran. The bogflow initially became known as the Dawn of Hope landslide (e.g. Petley 2020) but in this paper it is identified as ‘BBM-20’ (i.e. ‘Boleybrack Mountain 2020’). A video showing the entire extent of affected land can be viewed on YouTube (https://www.youtube.com/watch?v=ttJsyGSBPcc – Flynn 2020). The source area is located on the south-facing side of a very broad and gently sloping spur at the southeastern end of Boleybrack Mountain (Fig. 2a), at around 270 m above sea level. The underlying geology is the Gowlaun Shale Formation of Carboniferous (Namurian) age, comprising silty sideritic shale (GSI 2021). Much of Boleybrack Mountain is a designated Special Area of Conservation with active blanket bog being one of the five habitats that led to this status (NPWS 2013). Commercial forestry has encroached upon the southern tip of the Boleybrack upland in recent years. The 2004 edition of the topographic map shows no forest within 500 m of the failure site (OSI 2004), but at the time of the failure there was young plantation forest immediately north, west and south of the bogflow’s source area (Flynn 2020). The trigger for the failure was most likely localised very heavy—though perhaps not ‘extreme’—rainfall on 28 June 2020, with 44.4 mm recorded at Finner Camp, Co. Donegal (33 km to the NNW) (Met Éireann 2020a) and Newport, Co. Mayo, experiencing its wettest June day in its 60-year record on the same day with 53.4 mm (Met Éireann 2020b). This followed an unusually warm and dry second quarter of the year.

#### Characteristics of the landslide

Dykes and Warburton (2007b) defined a ‘bogflow’ as ‘Failure of a *blanket bog* (i.e. bog peat) involving the break-out and evacuation of semi-liquid highly humified basal peat from a clearly defined source area’ (p.79, their italics). The morphology of a bogflow is very distinctive: BBM-20 is an excellent example of the type (Fig. 2). The defining characteristic is that most of the source area perimeter comprises large (e.g. up to 15–20 m long, up to 3–5 m wide and typically > 1 m thick) intact blocks or ‘rafts’ of peat that have been displaced downwards into the source area by vertical subsidence, back-tilted rotation and/or occasionally forward toppling, often with a crescentic pattern (Figs. 2b, c). In this case, the toe of the source area appears to coincide with the head of the tributary stream course adjacent to a narrow strip of forest (Fig. 2d). Upstream of this point, the 1:50,000 map (OSI Sheet 26) shows a steepest average gradient (over ~ 350 m distance) of no more than 3.3°, but the gradient into the tributary valley varies between around 3 and 6° suggesting a convex change of slope that is significant at such low gradients.

The thickness of the peat is not known but measurements at other failure sites (e.g. Dykes and Jennings 2011; Dykes 2019) suggest that the base of the peat is likely to be up to around 3 m deep, and possibly more, across much of the affected area at BBM-20. Based on the video of Flynn (2020) and the (pre-failure) GE view of the site, the source area is around 450 m long and up to 250 m wide, with a minimum volume of 200,000 m^3^ if the peat is slightly less than 3 m deep throughout. The properties of the catotelm (i.e. lower, permanently saturated anaerobic layer below 0.5–1.0 m depth) of Irish blanket peat are summarised in Table 1. Based on these, this bogflow released many thousands of tonnes of solids, mostly comprising colloidal fragments of highly humified (decomposed) amorphous remains of plant matter, which were carried by the runout flow and contaminated watercourses and farmland up to 7 km downstream. However, much of the displaced peat, including many large rafts, remained within the source area.

**Table 1:** Values of peat properties, measured in the laboratory, of undisturbed samples obtained from near the base of the (lower catotelm) peat in the source area margin at each landslide

Property of peat	Units	Range	No. of bogflows	No. of bog slides	Reference(s) ^a^
Water content (field-wet)	% (mass fraction)	450–990	5	3	A, B, C, D
Water content (saturated)	% (mass fraction)	450–1160	5	3	A, B, C, D
Liquid limit	% (mass fraction)	~ 700–800	2	1	A
Volumetric water content (saturated)	Ratio	0.88–0.97	3	2	A, B
Bulk density (saturated)	kg m^−3^	1010–1120	5	3	A, B, C, D
Bulk density (dry)	kg m^−3^	100–200	5	3	A, B, C, D
Ash content	%	1.0–5.5	5	3	A, B, C, D
Humification	von Post scale	8–10	3	2	A, B
Saturated hydraulic conductivity (vertical)	m s^−1^	< 10^−11^–10^−5^	2	3	A, C
Saturated hydraulic conductivity (horizontal)	m s^−1^	10^−11^–10^−6^	3	2	B, C, D
Undrained shear strength (triaxial)	kPa	1.6–2.4	3	0	D
Undrained shear strength (direct simple shear)	kPa	5.0–6.0	0	0	E ^b^
Tensile strength	kPa	< 3 (< 4)	3	0	D, E ^b^

^a^A = Yang and Dykes (2006), B = Dykes (2008c), C = Dykes et al. (2008), D = Foteu Madio and Dykes (2018), E = Dykes (2019)

^b^Data from a peat slide in Co. Antrim

There are several features that indicate what probably happened here, partly in line with Petley’s (2020) preliminary observations:

1. Upslope of the convex break of slope (near the bottom of Fig. 2d), all parts of the source area margins show tension cracking of the upper peat as it was dragged inwards and downslope by the lower peat, whereas downslope of this point both sides have been impacted by moving peat (the flow and intact floating rafts) from upslope. This suggests that the failure originated at, or upslope of, the convex break, with everything further upslope developing by retrogression. This is a common scenario as observed at 8 out of 15 previous bogflows, where very deep and very soft and wet peat on low-angle topographic benches on the substrate exerts stress onto slightly thinner, drier and firmer peat on the adjacent steeper slope. The other 7 of the 15 occurred at the edges of escarpments where the same configuration is more pronounced.
2. There is a main source area which extends directly from the toe to the forest plantation, within which almost all of the surface peat has been carried away by the flow. The ‘main’ head extends back into the forest for a short distance. Figure 2c shows the trees—and therefore the pre-forestry drainage plough lines—to lead directly towards the location of the bogflow. This would have preferentially directed the rainwater into the deep peat on the slope outside the forest, especially if the peat contained well-developed pipe networks as observed elsewhere (Holden and Burt 2003; Warburton et al. 2004; Dykes et al. 2008), contributing to the lateral loading throughout the lower peat against the break of slope at the toe. Conversely, further headward retrogression was probably prevented by root reinforcement of the acrotelm peat by the trees.
3. There is an extensive area of upslope retrogression to the east of the main source area where the lower peat has failed but was mostly unable to fully mobilise and flow down the slope. However, a train of trees leading away from the ‘secondary’ head of the bogflow (Fig. 2b) shows significant displacement of rafts of upper peat floated on a narrow channel of flowing lower peat. This suggests that there may be a topographic drainage channel along the mineral substrate surface, within which the peat may always have been extremely wet and possibly already semi-liquid in consistency. The large rainwater inputs from the forestry drains can be expected to have exerted very high and possibly artesian water pressures around this channel (as observed elsewhere: Dykes and Warburton 2008), promoting the very localised flow and its own lateral retrogressive margins.

### MBW-20b: Meenbog Windfarm bogflow

#### Site characteristics and trigger conditions

The source area of this bogflow (Fig. 5) is at 54°43ʹ06ʺN, 7°52ʹ30ʺW (Ordnance Survey Ireland grid coordinate H 081 856), approximately 8 km southwest of Ballybofey and 5 km east of the Barnesmore Gap, Co. Donegal, 700 m west of the international border with the UK (Northern Ireland). Two videos of this site can be viewed on YouTube: one is an aerial drone footage from the distal runout to the source area (https://www.youtube.com/watch?v=xbQe55YnW5g: Derg Media 2020), while the second (https://www.youtube.com/watch?v=rf6S9Uz2Zrw: Donegal Daily TV 2020) shows the flow passing the truncated road at the exit from the source area, which must be only a short time after the initial failure. The source area is located at around 265 m altitude on a small spur of Carrickaduff Hill that descends roughly northeastwards towards Meenbog. The underlying geology comprises Precambrian metamorphosed sandstones (GSI 2021).

The location of the failure site is within a large area of commercial forestry, but the main source area developed within a rectangular parcel of unplanted ground roughly 380 m N-S × 280 m E-W. It is not known why this plot was never planted. The regular geometry suggests a land ownership issue but it may be that the peat was known to be too soft and weak and/or too deep to support any forestry operations. The Meenbog Windfarm project led to felling of trees within many similar parcels of land along with construction or upgrading of a dense network of access roads/tracks, including ‘floating roads’, to facilitate construction of the turbines. AGEC (2017, p. 12) noted that the pre-existing floating roads varied in type but commonly comprised ‘tree brash/trunks laid directly onto the peat surface and/or … geogrid overlain by up to 500 mm of coarse granular fill’. Note that there are no turbines within 300 m of the landslide source area, nor within 200 m of the runout path, and therefore, that turbine base construction works are not relevant to the peat failure.

Site investigations by AGEC (2017, p. 12) found peat depths along the proposed new access roads to be ‘typically less than 3.0 m with localised depths of up to 4.5 m recorded’. The very low gradient of the unplanted plot and its position at the foot of a much steeper slope could be expected to give rise to a very deep and very wet accumulation of peat. Peat more than 3.0 m deep was recorded around a road within 60 m NW of the unplanted plot, and in the area of the eventual failure, a road identified as a ‘new access track’, along the eastern edge of the same plot, crosses peat 2.5–3.0 m deep (AGEC 2017). The general susceptibility of the blanket peat to failure, as throughout Ireland, is highlighted by the occurrence of the large Barnesmore bogflow in November 1963 (Colhoun et al. 1965) on the same tract of upland just 3.5 km further northwest.

**Fig. 3:**
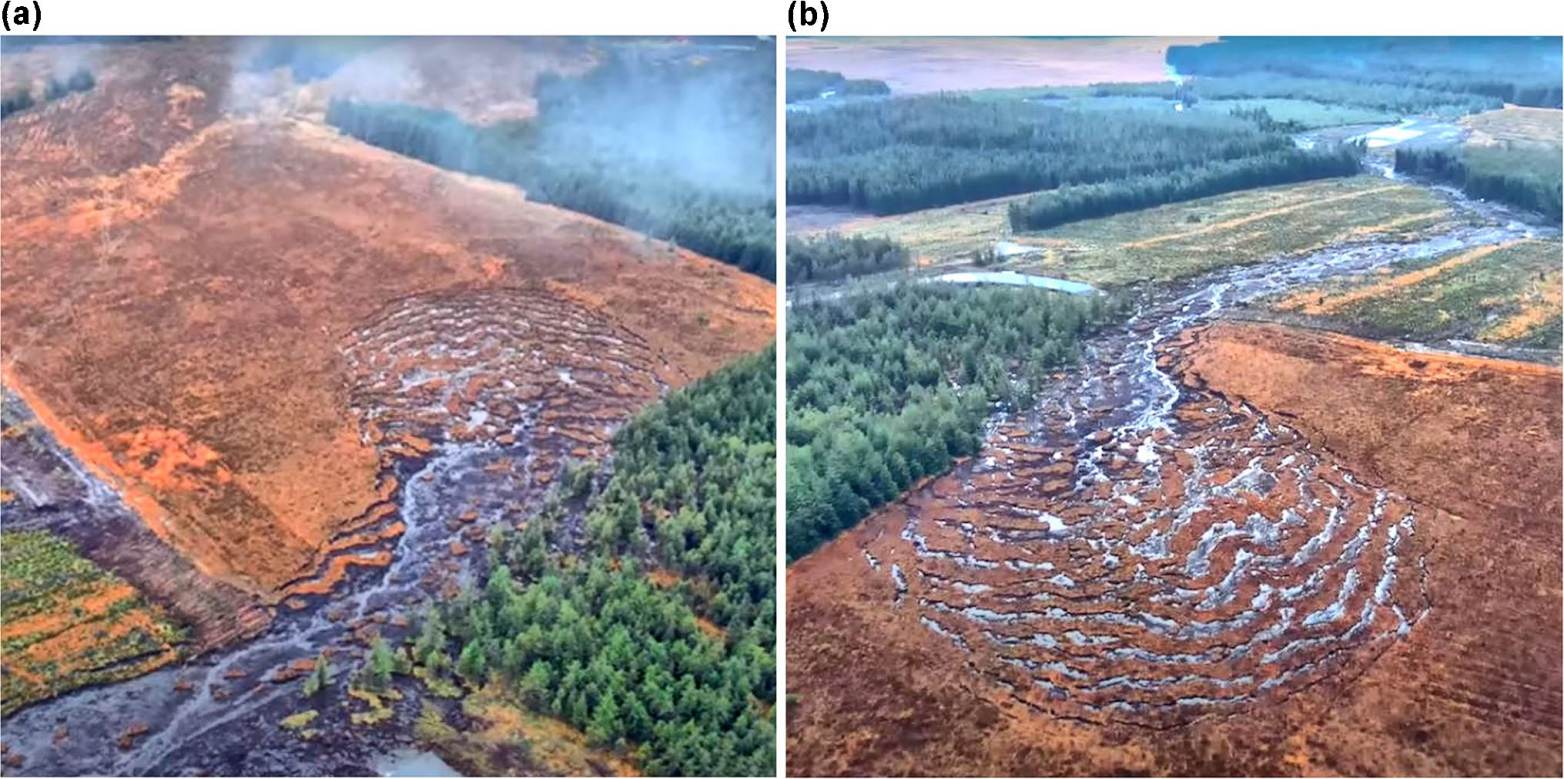
The source area of the Meenbog Windfarm bogflow. a View towards the SW showing the steeply rising slope at the far end of the unplanted plot (top left). Source: 0 m 47 s in Derg Media (2020). b View towards the NE showing the site of the initial peat flow (beyond the new access road) and the runout extending into a natural drainage line. Source: 1 m 10 s in Derg Media (2020)

The rainfall recorded at Finner Camp, the nearest meteorological station to MBW-20, shows no evidence for a rainfall trigger any time between 1 and 13 (date of bogflow) November. However, the lower end of the source area, where the displaced peat effectively channelised into a runout track, is located where the ‘new access road’ crosses the eastern boundary of the unplanted plot. The drone video (Derg Media 2020) clearly shows that tree brash/trunks had been placed along the first 60–70 m along the plot boundary and that the gravel fill was being advanced towards the corner of the plot (bottom left corner of Fig. 3a). Therefore it appears likely that the failure is associated with construction of this new access road, as discussed below.

#### Characteristics of the landslide

The morphology of the source area of MBW-20b—upslope of the new access road—is another excellent example of a bogflow with the same general characteristics as described for BBM-20 (Fig. 3). The inward tearing of rafts of peat from the lateral margins, including from the adjacent forested plot (Fig. 3b), indicated that the failure began downslope of these zones—i.e. at or near the new access road. Note the limited expansion into the forested area compared with the open plot. The 1:50,000 map (OSI Sheet 11) suggests a gradient of little more than 1° across the open plot and around 3.8° downslope from the road. The peat depth throughout the source area can reasonably be assumed to be at the upper end of the measured 2.5–3.5 m range (see above). Based on the video of Derg Media (2020), the source area upslope from the road is around 240 m long and up to 120 m wide. A mean peat depth of slightly less than 3 m gives the failure a volume of 65,000 m^3^. The catotelm peat can be reasonably expected to have properties consistent with Table 2. Therefore, this bogflow also released several thousand tonnes of polluting solids in addition to runout from the initial peat flow.

**Table 2:** Frequency of known incidence of common features of failure sites

**Failure site characteristics** ^a^	**Bogflows (out of 17)** ^b^	**Bog slides (out of 20)** ^b^
Edge of escarpment	7	*None*
Concave break of slope	*None*	6^c^
Convex slope/convex break of slope	8 plus **BBM-20, MBW-20b**	6 plus **KFM-20** ^d^
Stream channel at toe of failed slope	***BBM-20**** – but not like the bog slides*	4
One or more drainage or boundary ditches	8	5
Forestry plough lines and/or ditches	2 plus **BBM-20** (*possibly ****MBW-20b***)	2 plus **KFM-20**

^a^Two failures were associated with peat cutting (both manual and by tractor-mounted Difco (‘sausage’) machine, and two other failures may or may not have been affected by manual peat cutting

^b^Totals include the three 2020 landslides

^c^All bog slides with concave breaks of slope have length:width ratios ≤ 2.4 (except BCF-88) and volumes ≤ 27,000 m^3^

^d^All bog slides on convex slopes have length:width ratios ≥ 6.4 and volumes ≥ 45,000 m^3^ (+ SBO-73 with 19,000 m^3^)

**Fig. 4:**
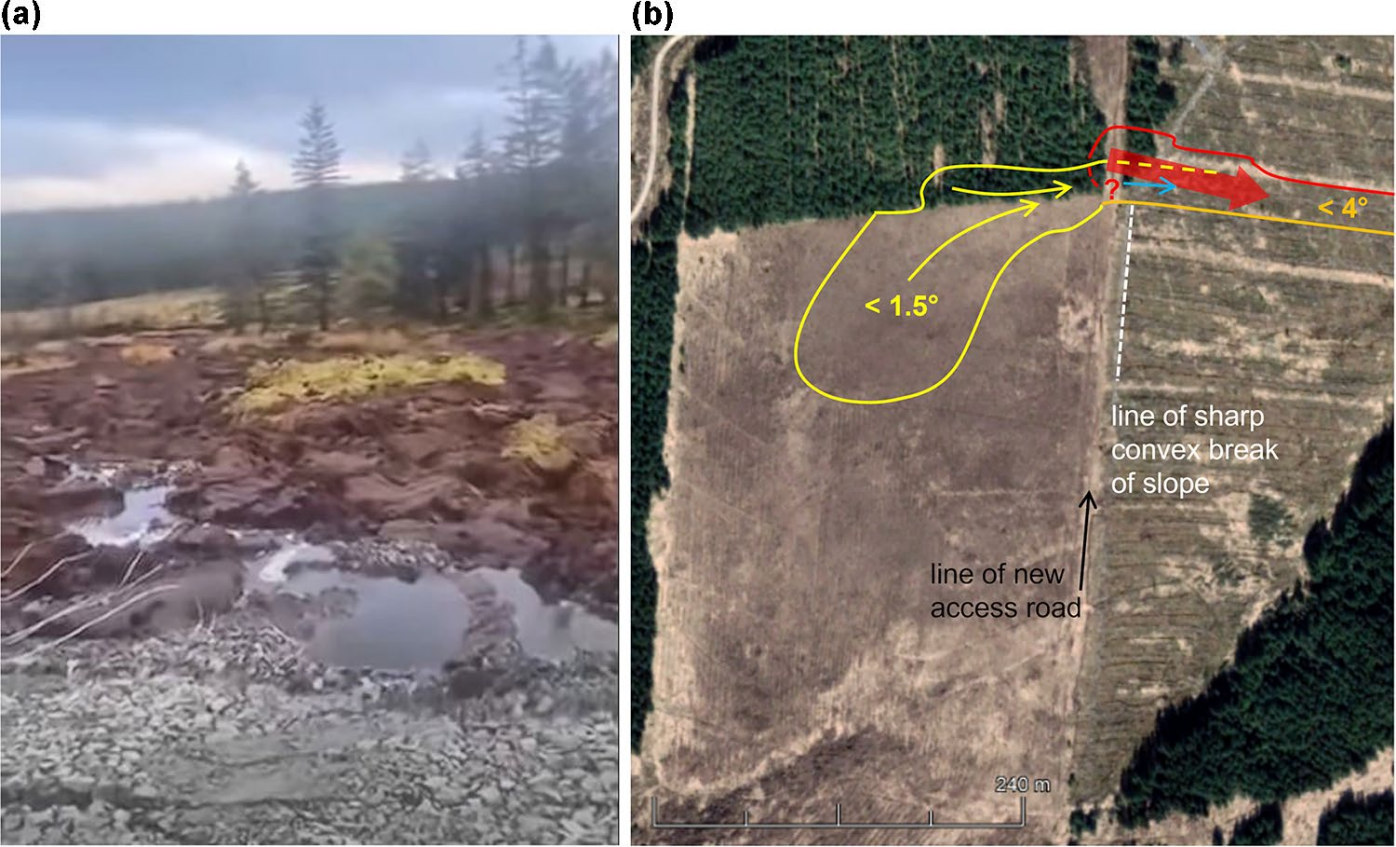
**a** The head of the initial peat flow with the collapsed end of the gravel road in the foreground and trees floating past from right to left around 40–50 m ahead. Source: 0 m 26 s in Donegal Daily (2020). **b** Outline plan of the site: red = initial peat flow, yellow = bogflow, orange = southern margin of both failures, light blue arrow = moving trees in the video of the bogflow. Note the steep slope rising from the unplanted plot at the bottom of the image

There are several pieces of visible evidence in the Donegal Daily (2020) video that strongly suggest the bogflow to have been indirectly caused by the access road construction, by means of a preceding failure. Firstly (0:26–0:34 in the video: Fig. 4a), the furthest part of the advancing gravel fill for the new access road can be seen to have subsided into an area of collapsed peat, with some small patches of gravel visible several metres downslope from the road at 0:34. Secondly, the peat ahead of, and downslope from, this collapsed end of the road is entirely disrupted, broken into chaotic blocks. This is typical of ‘peat flows’ (Dykes and Warburton 2007b) triggered by head-loading, as observed at other known examples (e.g. Slieve Bearnagh – Dykes 2008b; Ballincollig Hill – Dykes and Jennings 2011). Thirdly, this zone of chaotic blocks is stationary, i.e. an initial head-loaded failure has ceased, but trees from the edge of the bogflow source area, less than 100 m further upslope, are just starting to float past this point standing on intact rafts of peat. This is a strong indication that an initial peat flow at the convex break of slope (Fig. 4b) removed lateral support for, and thus allowed the release of, very wet and weak lower catotelm peat from the unplanted plot; i.e. the bogflow constitutes a separate second failure.

It is not clear whether the initial failure (‘peat flow MBW-20a’) involved the full width of the gap in the new access road (around 60 m) or, as the two videos seem to suggest, just the ~ 30 m nearest the collapsed gravel section (Fig. 4b). It is also not clear how far downslope the initial failure of peat extended, although there appears to be significant loss of peat from an erosion track around 40 m wide extending to the drainage line 290 m further downslope. If the peat here is 2.5 m deep, then—including the peat flow head near the road—at least 30,000 m^3^ of peat may have been displaced within this zone, probably sending at least several hundred tonnes of polluting solids downstream.

### KFM-20: Knockanefune Mountain

#### Site characteristics and trigger conditions

**Fig. 5:**
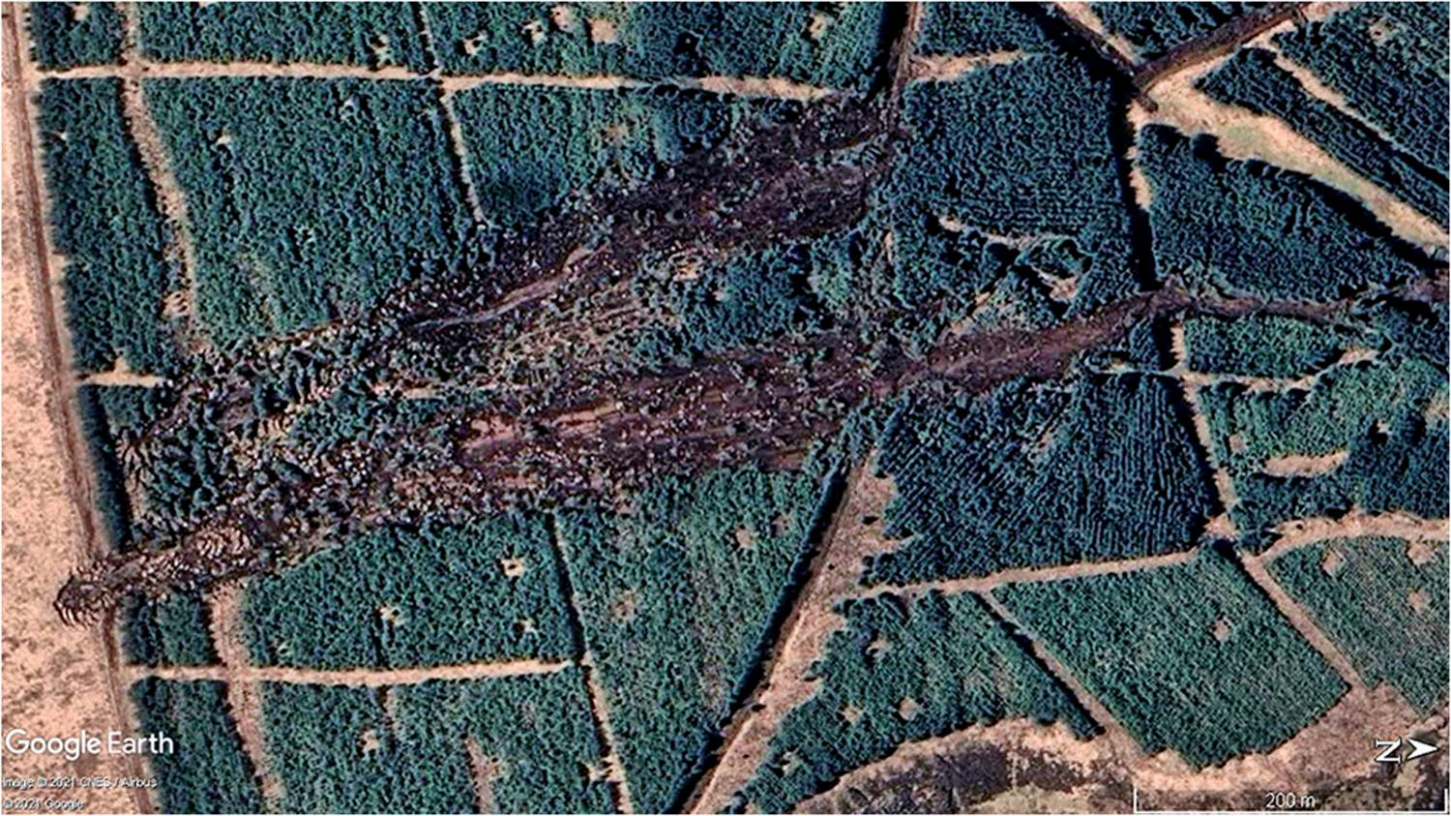
The ‘Mount Eagle’ bog slides on Knockanefune Mountain. KFM-20 is the lower slide with a head extending into the open blanket bog above the forest (lower left). The straight, narrow track around 1/3 of the way down the slope coincides with the main convex break of slope, where distinct failure surfaces are visible in both landslides. Source: Google Earth (Image © 2021 CNES/Airbus; ©2021 Google)

The source area of this bog slide is at 52°13ʹ30ʺN, 9°18ʹ12ʺW (Ordnance Survey Ireland grid coordinate R 110 090), 11 km east of Castleisland, Co. Kerry. It is located on the north-facing slope of the roughly east–west mountain ridge immediately southwest from Knockanefune Mountain, 2 km southeast from Mount Eagle, at around 430 m above sea level. Two videos of this site can be viewed on Twitter: one is an aerial drone footage of the mid-lower source area (https://mobile.twitter.com/savekerry/status/1328059773825257475?lang=en-GB: Save Kerry 2020a), while the second (https://twitter.com/savekerry/status/1328342795795492866?lang=en: Save Kerry 2020b) shows some views within the source area from the ground. The underlying geology is the Feale Sandstone Formation of Carboniferous (Namurian) age, comprising alternating sandstone with shale/siltstone (GSI 2021). The location is within a private forest plantation, which is shown on the 2006 edition of the topographic map (OSI 2006). The new bog slide formed alongside a near-identical failure dating from sometime between 27 March 2012 and 29 March 2019 (dates of GE images). Both landslides are visible on the latest (22 April 2021) GE image of this location (Fig. 5). The exact date of this bog slide is not known but it was shortly before the Meenbog event (Save Kerry 2020a, b). Rainfall records from the nearest Met Eireann stations show a significant (but not exceptional) widespread rainfall event on 11 November with very high antecedent rainfall, which seems to be the most likely trigger.

#### Characteristics of the landslide

**Fig. 6:**
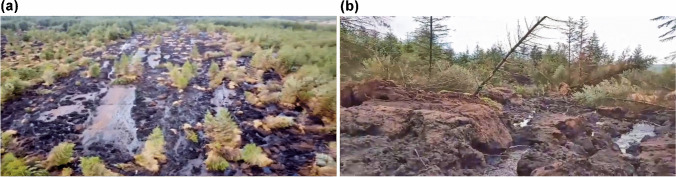
**a** View down the latest bog slide KFM-20 from just above the convex break of slope. The older slide can be seen at the extreme upper left. Source: 0 m 34 s in Save Kerry (2020a). **b** Displaced rafts and blocks of peat within the lower part of the source area. Source: 0 m 06 s in Save Kerry (2020b)

Dykes and Warburton (2007b) defined a ‘bog slide’ as ‘Failure of a *blanket bog* (i.e. bog peat) involving sliding of intact peat on a shearing surface within the basal peat’ (p. 81, their italics). The morphology of a bog slide can be variable between that of a bogflow and a peat slide, but in general the lateral margins tend to be more clearly defined (fewer subsided strips or ‘rafts’ of intact peat). The forestry context makes it more difficult to see the features clearly, but some extensive areas of basal shear surface can be seen in Fig. 6a and the geometry is essentially long and linear although widening slightly in the downslope direction. The upper 250 m length, above the 400 m contour, is almost planar at around 5.8°; then, the slope steepens to around 10.8° (1:50,000 map, OSI sheet 72).

Figure 5 appears to show that KFM-20 developed throughout most of its length at the same time, given that there are effectively no strips or rafts of peat formed by tensile stresses at the margins right up to the head margin. The high density of peat rafts stranded within the upper 150 m can be attributed to basal sliding friction on the lower gradient retarding their movement. Figure 5 also shows that the upper part of the older landslide may have formed by retrogressive expansion above the break of slope, indicated by the much wider head zone above a narrow ‘neck’. This would suggest that failure initiated somewhere around the break of slope, probably therefore in both cases given the similarity of the context and, most likely, the peat and condition of the forest.

The depth of peat is unknown, but the blocks and rafts of peat visible in the videos (e.g. Figure 6b) appear consistent with peat up to 2 m deep although probably less on the steeper lower part of the slope. The total length of the source area is around 480 m and the width increases from ~ 30 m at the head to ~ 60 m throughout the lower half. If the depth of peat is assumed to be 2 m at the head and 1.5 m on the lower slope, then the total failure volume is around 40,000 m^3^. Again, at least several hundred tonnes of solids are likely to have reached the natural watercourses with much of this eventually polluting River Clydagh.

## Discussion

### Comparisons with previous bogflows and bog slides

**Fig. 7:**
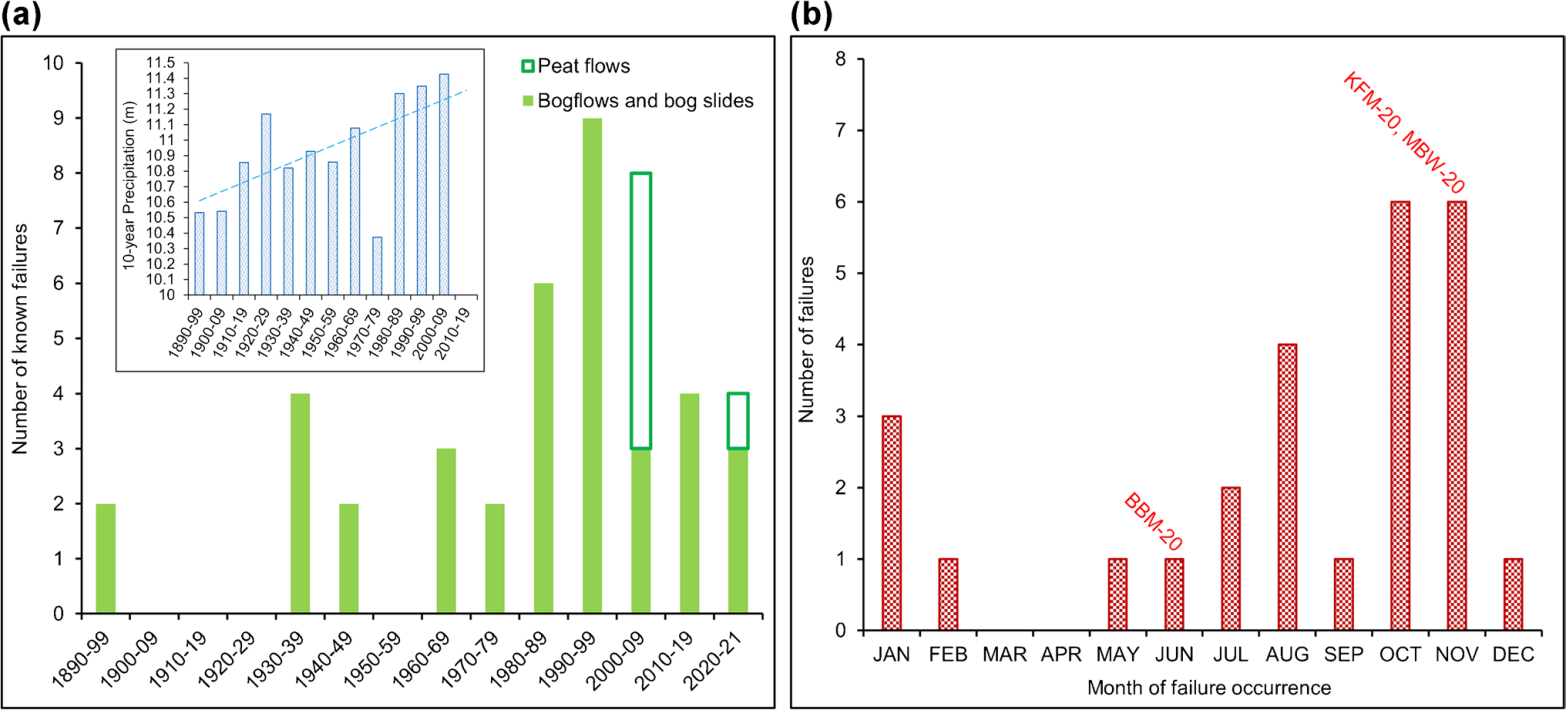
Occurrence of reported bogflows and bog slides in Ireland. **a** Frequency by decade. Peat flows are also shown to highlight the period of accelerated onshore windfarm developments leading to greater awareness and regulation of the risks. Inset: Decadal rainfall totals for the Republic of Ireland (Met Eireann 2020b). The data for 2010–2019 are not yet available. **b** Seasonal occurrence of bogflows and bog slides

**Fig. 8:**
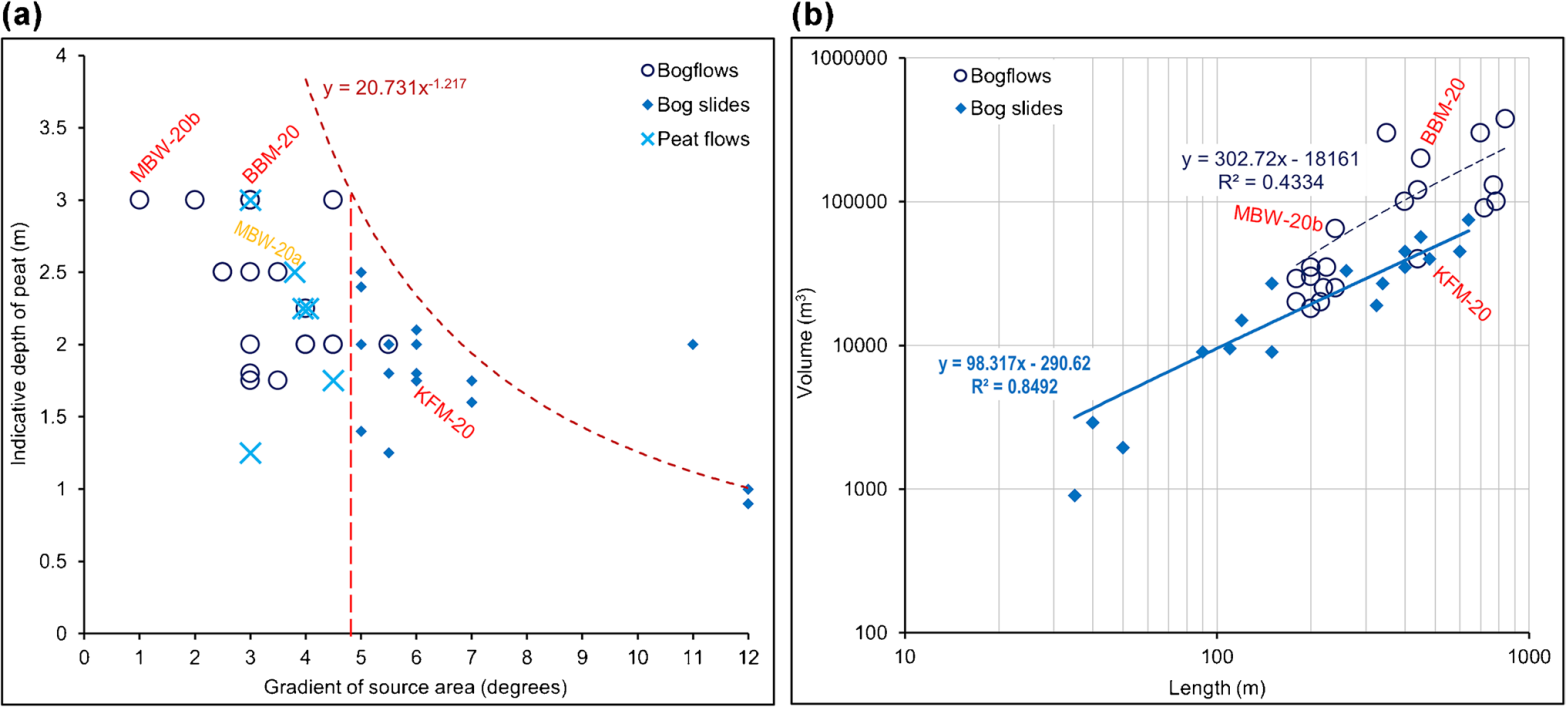
Geometrical characteristics of Irish bogflows and bog slides, showing how the 2020 landslides compare with earlier examples. **a** Gradient vs. estimated peat depth showing an empirical threshold from Co. Mayo (after Dykes and Warburton 2007a). **b** Volume of displaced peat vs. centre-line length of source area

Despite the small sample size (even for Ireland), some distinct patterns can be identified that provide context for the events of 2020 reported above:

1. *Occurrence—*The frequency of occurrence of these large blanket bog failures appears to have been increasing throughout the last 130 years—although the average remains just below three per decade. This should perhaps be viewed in the context of apparently increasing annual rainfall for Ireland (Fig. 7a), although there is no significant statistical relationship. For the failures with known dates of occurrence, a higher frequency associated with higher autumn rainfall is clearly indicated in Fig. 7b but very heavy summer rainfall following prolonged hot and dry periods—predicted to become a more common scenario—is a recognised risk factor. The overall pattern has shifted 2–3 months earlier compared with a similar analysis by Alexander et al. (1985). The BBM-20 bogflow is the first example known to have occurred in June but changing rainfall patterns may be expected to give rise to more in future years.

2. *Site characteristics—*Several commonly occurring features of bogflow and bog slide failure locations have been noted in previous published accounts of specific examples, and reviewed for all types of peat failures by Dykes and Kirk (2006) and Dykes (2008a). These are summarised in Table 2. The ‘drainage or boundary ditches’ include all types of hydrological and/or structural influences of cut or excavated features, but ditches and ploughing for forestry planting are considered separately. The different topographic contexts associated with the two failure types are worthy of note and the three recent landslides can be seen to have features common to their respective types.
3. *Topography—*The relationship between gradient of slope and depth of peat is thought to be a key factor in understanding blanket bog failures. Figure 8a shows peat depths corresponding with dominant gradients at all the landslides examined for this study. Two things are readily apparent. Firstly, bogflows tend to be associated with slightly deeper peat on lower gradient slopes than bog slides, and that the two groups of failures appear to be separated by a threshold slope of 4.75°. Secondly, with one exception (the Moanbane bog slide: Mitchell 1938), a peat depth-gradient curve obtained from 32 landslides on Dooncarton Mountain, Co. Mayo (Dykes and Warburton 2007a) appears to define an upper limit for these types of failures. Although MBW-20b has an unusually low gradient, the three recent failures are otherwise consistent with the previous recorded examples.
4. *Geometry—*Several simple geometrical parameters were examined to determine whether any other relationships existed that could help to explain the different characteristics of bog slides and bogflows. In both types of landsides, deeper peat generally allows greater volumes of peat to fail but the relationships differ. Figure 8b shows that bog slides tend to be smaller and that bogflows can develop to be extremely large, with the larger bog slides (> 200 m long) being 4½ to 8½ times longer than their maximum widths while most bogflows show much greater lateral expansion relative to their lengths. No other relationships could be identified. Only the gradient showed a clear threshold dividing the two groups.
5. *Peat characteristics—*Ash content, mass fraction water content and field-wet or saturated bulk density are key indicators of the in situ characteristics of the peat (Hobbs 1986; O’Kelly 2015). Even the qualitative scale of peat humification determined by the von Post ‘squeeze test’ provides a degree of information that engineers find useful for characterising peat (Hobbs 1986; Carlsten 1993). Quantitative data from blanket bogs are now providing a basis for investigating the geotechnical properties and behaviour of peat in new and more detailed ways to improve our quantitative assessment of in situ peat strength (e.g. O’Kelly 2017) and understanding of mechanisms of failure. Table 1 presents data obtained from lower catotelm peat, i.e. the assumed failure zone, at five bogflows and three bog slides plus a recent ‘peat slide’ in Co. Antrim (Dykes 2019). It shows that the characteristics of lower catotelm peat that may relate to in situ strength seem to be broadly consistent throughout the island of Ireland (Dykes 2019), and are probably representative of the peat at all of the three recent landslides.

### Implications for peat failure hazard assessment

The three recent peat landslides seem to reinforce known common risk factors for the respective types of failure, in particular the possibility of very large bogflows from very low gradient (< 5°) convex slopes and smaller, probably less mobile bog slides from steeper slopes—although both types can deliver hundreds or thousands of tonnes of polluting peat particles to watercourses and so cause potentially significant economic as well as ecological damage for many kilometres downstream. Climate change predictions of warmer, drier summers but with a generally higher frequency of high magnitude rainfall events may be expected to cause more such landslides in the spring or summer than has been the case over the last century. Figure 7a suggests an increasing frequency of these types of failures but this may reflect greater scientific and management interest in recent years with more detailed and complete reporting of events.

All three of the landslides in this study involved blanket bog subjected to commercial forestry to some extent. A previous study suggested that mature forests provide a significantly reduced susceptibility to failure of the peat due to the combination of root reinforcement and drying of the peat by evapotranspiration processes (including interception losses) as a forest grows and matures (Dykes 2012). However, pre-forestry drainage and deep ploughing leave deep peat susceptible to failure from concentration of diverted runoff at certain slope locations or zones (Hendrick 1990), at least for the first few years while the peat remains substantially saturated but structurally weakened by the interventions. Cross-slope ditches associated with forestry sites, including drainage for the roads, may create longer lasting weaknesses, effectively removing toe support for the peat upslope of the excavations. This may have been a contributory factor for KFM-20, as observed elsewhere (e.g. Lindsay and Bragg 2005), with the reinforcing effects of the mature forest being overcome by the developing instability on the much steeper lower part of the slope. At BBM-20, it seems likely that forestry plough lines and drains passing a high volume of excess runoff towards an area of deep blanket bog constituted a significant causal factor, but that further retrogression of the failure was restricted by either the root networks or possibly because the peat mass was not continuous. At Meenbog, forestry drainage probably had little influence on the initial head-loaded peat flow (MBW-20a) due to low antecedent rainfall, but the net effect of the forest was to limit the lateral expansion of the subsequent bogflow (MBW-2b). Attention should perhaps be focused on reviewing the susceptibility to failure of intact blanket bogs outside the downslope edges of plantations.

## Conclusions

The occurrence of three large bogflows and/or bog slides in a single year is exceptional given the average of 0.3 such (reported) events per year over the last 130 years. Two were most likely triggered by high but not exceptional rainfall, and the third appears to have been initiated by a separate head-loaded peat flow associated with construction of a windfarm access road. Remote investigations were made possible by the current trend of ordinary people to obtain photos or videos of unusual things and to post them on-line to be viewed by anyone. Examination of such non-field evidence suggests that all three landslides have site characteristics that are consistent with known risk factors, particularly convex breaks of slope but also the structural and hydrological effects of forestry plough lines and ditches. At BBM-20, these forestry modifications to the bog probably contributed hydrologically to the occurrence of the failure, and possibly structurally at KFM-20, but at the same time further retrogression of BBM-20 and MBW-20b into adjacent forest may have been retarded by the preventative effects of root networks and evapotranspiration. Finally, the geometric characteristics of the source areas are consistent with the reported ranges of values from previous failures. As such, there do not appear to be any novel or unexpected features among the three recent landslides.

## Data Availability

Data sharing is not applicable to this article as no datasets were generated during the current study.
